# Tranexamic acid decreases rodent hemorrhagic shock-induced inflammation with mixed end-organ effects

**DOI:** 10.1371/journal.pone.0208249

**Published:** 2018-11-29

**Authors:** Patrick F. Walker, Anthony D. Foster, Philip A. Rothberg, Thomas A. Davis, Matthew J. Bradley

**Affiliations:** 1 Department of Regenerative Medicine, Naval Medical Research Center, Silver Spring, Maryland, United States of America; 2 Department of Surgery, Uniformed Services University and Walter Reed National Military Medical Center, Bethesda, Maryland, United States of America; Ludwig-Maximilians-Universitat Munchen, GERMANY

## Abstract

Beyond its anti-fibrinolytic mechanism, tranexamic acid has been suggested to have anti-inflammatory properties which may contribute to the survival benefit it provides to trauma patients. The objective of this study was to assess possible immunomodulatory effects of tranexamic acid as well as potential amelioration of end-organ injury in a rodent hemorrhagic shock model. Controlled hemorrhagic shock was induced in adult Sprague Dawley rats to a mean arterial pressure of 30 mmHg. Groups of 10 rats were administered intravenous tranexamic acid (300mg/kg) or vehicle control (normal saline) intravenously 15 minutes after the induction of shock. After 60 minutes of hemorrhagic shock, resuscitation was started. Animals were euthanized at six, 24, or 72 hours from the start of shock. Serum laboratory values to include inflammatory biomarkers were measured, and end organ histology was evaluated. Tranexamic acid treatment was associated with a significant decrease in serum IL-1β at six and 24 hours and IL-10 at 24 hours from start of shock compared to vehicle control. Histologic analysis demonstrated mild decreases in both perivascular pulmonary edema and follicular mesenteric lymph node hyperplasia in the tranexamic acid treatment group but also increased myocardial lymphocytic infiltration with necrosis and degeneration. Tranexamic acid was also associated with a small but significant increase in peripheral neutrophil count as well as a significant decrease in neutrophil aggregation in pulmonary tissue at six hours post-injury. These data thus demonstrate a mixed effect of tranexamic acid. While there was an improvement in pulmonary edema and a suppressive effect on several key inflammatory mediators, there was also increased myocardial degeneration and necrosis, which is possibly related to the pro-thrombotic effect of tranexamic acid.

## Introduction

Hemorrhagic shock (HS) is a leading cause of preventable death in civilian and military trauma patients [1,2]. Tranexamic acid (TXA) is a pharmacologic adjunct that can be given to patients in or at risk for HS. TXA is a synthetic lysine derivative that prevents fibrinolysis by blocking the conversion of plasminogen to plasmin [3]. Previous studies have demonstrated the utility of TXA to decrease intraoperative blood loss and blood product requirements in elective surgeries [4,5]. In CRASH-2 and MATTERs, two more recent studies evaluating the effectiveness of TXA in trauma patients, TXA administration was associated with improved survival in civilian and military trauma patients, respectively [6,7].

While the CRASH-2 study reported a decreased rate of death attributed to hemorrhage in patients who received TXA, there was not a significant difference in the need for transfusion between treatment and placebo groups. The MATTERs study showed that there was actually a higher transfusion requirement in a matched cohort that received TXA as well as a survival benefit that began at 48 hours post-injury, a time point where the risk of death from hemorrhage is typically decreased [8]. These findings have contributed to speculation that TXA may have an alternative mechanism of action contributing to improved survival in trauma patients.

There is a growing body of evidence suggesting that this beneficial effect of TXA is related to its potential anti-inflammatory effects. Plasmin promotes an upregulation of the inflammatory cascade via activation of pro-inflammatory cells and induction of pro-inflammatory genes [9]. By inhibiting the conversion of plasminogen to plasmin, TXA may mitigate the inflammatory response associated with HS. Notably, in cardiac surgery patients who have been placed on cardiopulmonary bypass, TXA has been shown to diminish the systemic inflammatory response [10,11].

Recently, a US Department of Defense review committee published an assessment of knowledge gaps and research requirements regarding TXA in trauma [12]. The authors described the need for further data regarding the mechanism by which TXA benefits trauma patients as well as potential adverse effects. With that in mind, we sought to evaluate the efficacy of TXA in mitigating systemic inflammation and reducing the severity of end-organ injury in a rat model of HS. We hypothesized that TXA administration may attenuate the initiation and propagation of HS-induced systemic inflammation as well as end-organ injury.

## Materials and methods

### Animals

Young adult pathogen-free male Sprague Dawley rats (*Rattus norvegicus*; 268-542g, mean 396.7 ± 65.9g) underwent the study protocol (Taconic Farms, Germantown, NY). All animals were housed in clean plastic cages and kept on a 12-hour light/dark cycle with unlimited access to food (standard rodent chow) and fresh water *ad libitum*. The study protocol (16-OUMD-03S) was reviewed and approved by the Walter Reed Army Institute of Research/Naval Medical Research Center Institutional Animal Care and Use Committee in compliance with all applicable Federal regulations governing the protection of animals in research. There were 10 animals per treatment and control arm at the six, 24, and 72 hour intervals. The animals were anesthetized with isoflurane. Analgesia after the procedure was provided with buprenorphine administered every 12 hours.

### Hemorrhagic shock model

We used a previously-described rat model for pressure-controlled hemorrhagic shock [13]. After the rats were anesthetized with isoflurane, a cutdown was performed to expose the femoral artery and vein, which were then cannulated with PE-50 tubing. To achieve controlled HS, blood was removed through the arterial catheter at a rate of 0.3mL/100g/minute until a mean arterial pressure (MAP) of 30mmHg was achieved. The blood was removed aseptically into a syringe containing 100 units of heparin. Normothermia was maintained at 37.5 ± 0.5 °C using a rectal sensor to monitor core body temperature. Blood pressure, heart rate, pulse oximetry, and temperature were recorded every five minutes during the 60-minute shock period using a Philips MMS X2 monitor (Amsterdam, Netherlands). The rats were subjected to 60 minutes of controlled hemorrhagic shock, with blood being given back or withdrawn as needed to maintain a MAP of 30 mmHg during this time.

### TXA administration and resuscitation

After 15 minutes of shock, a single intravenous bolus of either normal saline (0.9% NaCl; vehicle control) or TXA (300 mg/kg) was slowly administered intravenously as a single bolus over a two-minute period to the rats via the femoral vein. TXA was given at this time to model the Tactical Combat Casualty Care guidelines for TXA administration as a part of prehospital care in casualties suspected to require a massive transfusion [14]. In previous rodent studies, a TXA dosing of 300mg/kg was found to be the maximum effective dose for hemorrhage control without a reported increase in complications [15–17]. Shock was maintained for a total of 60 minutes. Resuscitation was then started through the venous cannula with infusion of normal saline at a volume twice the shed blood volume infused over 30 minutes. This was followed by infusion of half the heparinized shed blood over the next 30 minutes. Resuscitation was completed with administration of normal saline at twice shed blood volume again for 60 minutes. These ratios were in accordance with a previously described rat model for resuscitation after hemorrhagic shock [13]. Physiologic parameters were recorded every 15 minutes during the two-hour resuscitation period. Ten animals per treatment group were euthanized at designated intervals at six, 24, or 72 hours from the start of HS.

### Blood analyses

Blood was drawn for laboratory analysis at the induction of HS and was used for the “pre-hemorrhage” sample. The remainder was drawn immediately before euthanasia by cardiac stick. An aliquot of EDTA-treated blood was evaluated for complete blood cell count (CBC) (Sysmex xt2000i Automated Hematology Analyzer, Kakogawa, Hyogo, Japan). Serum chemistry values were measured including blood urea nitrogen (BUN), creatinine (Cr), lactate dehydrogenase (LDH), and creatinine kinase (CK) (Vitros 350 Chemistry System, Rochester, NY). Changes in inflammatory cytokines and chemokines were analyzed using a Luminex 100 IS xMAP Bead Array Platform (Millipore, Billerica, MA). A six-plex assay was used to measure interleukin-1β (IL-1β), IL-6, IL-10, tumor necrosis factor-α (TNF-α), monocyte chemoattractant protein-1 (MCP-1), and macrophage inflammatory protein-1α (MIP-1α). Fluorescence-activated cell sorting (FACS) was performed to obtain flow cytometry data regarding peripheral and splenic leukocytes.

### Histology and pathology

Sections of heart, liver, lung, kidney, small bowel (ileum), mesenteric lymphatic tissue, spleen, skin, and muscle were harvested during necropsy and immediately fixed in 10% neutral buffered formalin, processed for paraffin embedding, serially sectioned (3–5 μm) onto glass slides, and stained with hematoxylin and eosin (H&E). All slides were evaluated and scored on a standardized ordinal scale by a veterinary pathologist blinded to the experimental groupings (0 = minimal; 1 = mild or <25% of tissue affected; 2 = moderate or 26–50% of tissue affected; 3 = marked or 51–75% of tissue affected; 4 = severe or 76–100% of tissue affected).[18] The slides were graded for evidence of end-organ damage including edema, inflammatory cell infiltrate and necrosis. Myeloperoxidase (MPO) immunohistochemical (IHC) staining was also performed on myocardial and pulmonary tissue to evaluate for neutrophil infiltration.

### Statistical analysis

Continuous parametric data were analyzed by a two-sample T test. Non-parametric continuous data were analyzed by a Wilcoxon rank-sum test. A *p-value* < 0.05 was considered statistically significant.

## Results

**Fig 1:**
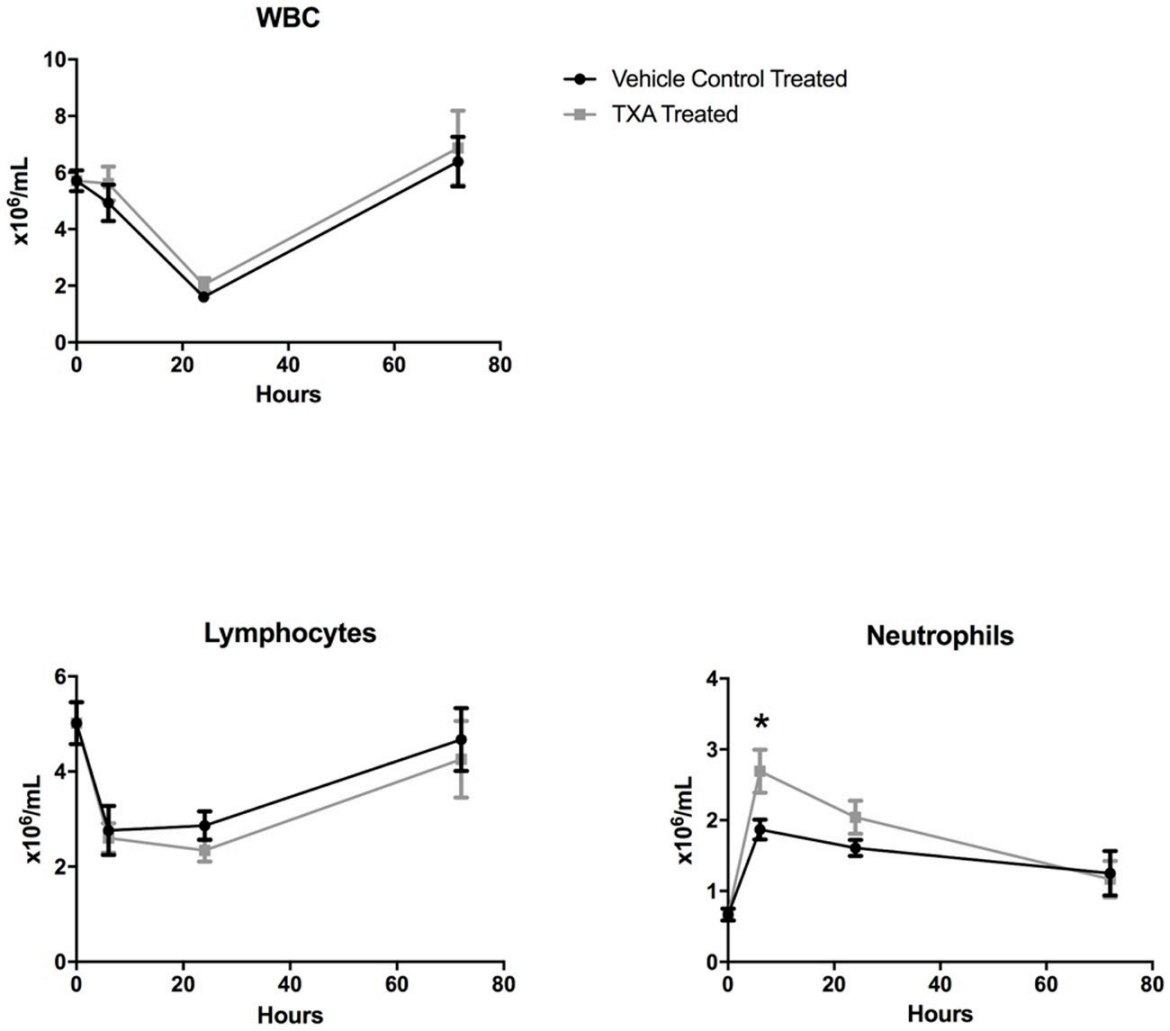
White blood cell (WBC) count with neutrophil and lymphocyte differentials in rats subjected to controlled hemorrhagic shock and resuscitation, vehicle control (normal saline) treated vs. tranexamic acid (TXA) treated, 300mg/kg (*p<0.05, error bars = SEM).

**Fig 2:**
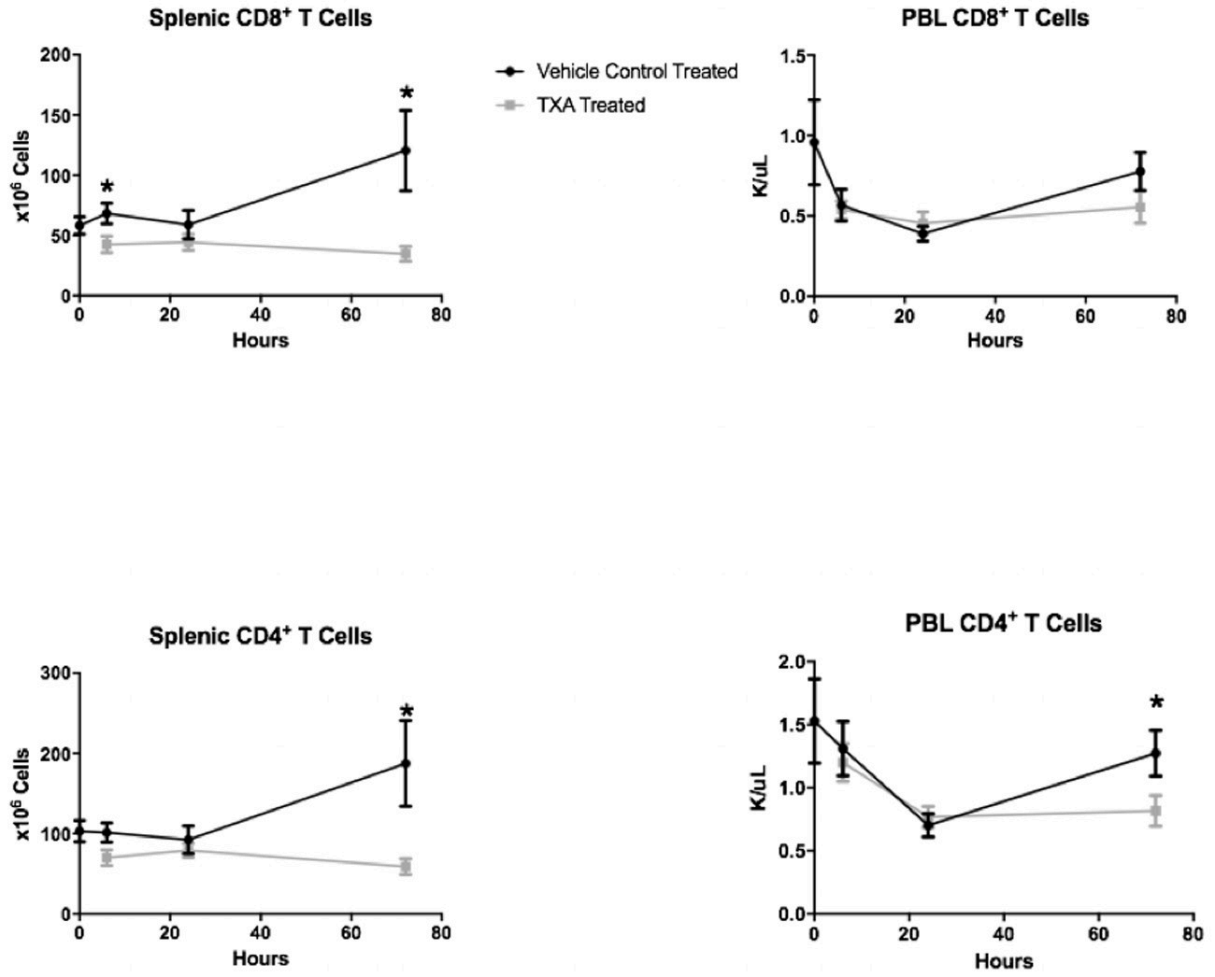
Fluorescence-activated cell sorting (FACS) analysis of splenic and peripheral blood (PBL) CD8+ and CD4+ T cell levels in rats subjected to controlled hemorrhagic shock and resuscitation, vehicle control (normal saline) treated vs. tranexamic acid (TXA) treated, 300mg/kg (*p<0.05, error bars = SEM).

Controlled HS was induced in the study animals by the removal of a mean of 13.5 ± 2.6 mL of blood to achieve the desired MAP of 30 mmHg. CBC analysis demonstrated that the total white blood cell (WBC) count as well as the lymphocyte count decreased in both treatment groups after the induction of HS (Fig 1). The nadir was at 24 hours, and there was no difference between the TXA and vehicle control treatment arms. The number of circulating neutrophils increased in both treatment arms, however, peaking at 6 hours after HS. The number of circulating neutrophils at 6 hours post-injury was 2.7 ± 1.0 x 10^6^/mL in the TXA treatment group compared to 1.9 ± 0.4 x 10^6^/mL in the vehicle control group (p = 0.03). On FACS analysis, TXA was associated with a decrease in splenic CD8+ T cells at 6 hours (42.6 ± 20.9 x 10^6^ vs. 68.2 ± 19.0 x 10^6^, p = 0.04) and 72 hours (34.8 ± 18.9 vs. 120.4 ± 88.2 x 10^6^, p = 0.01) post-injury. TXA was also associated with a decrease in splenic CD4+ T cells at 72 hours (59.1 ± 29.0 x 10^6^ vs. 187.4 ± 141.1, p = 0.02) as well as peripheral CD4+ T cells at 72 hours (0.8 ± 0.4 x 10^6^ vs. 1.3 ± 0.5 x 10^6^, p = 0.047) post-injury (Fig 2).

Rats in both treatment groups demonstrated increased BUN and creatinine levels after the induction of hemorrhagic shock compared to baseline (Fig 2). At 24 hours, the TXA treatment group had a significantly decreased BUN (19.8 ± 4.9 mg/dL) compared with the vehicle control treatment group (24.3 ± 5.1 mg/dL, p = 0.02). LDH and CK increased after the start of hemorrhagic shock with peak levels at 24 hours and 6 hours, respectively, post-injury. There was no statistically significant difference in LDH or CK levels between the two treatment groups.

**Fig 3:**
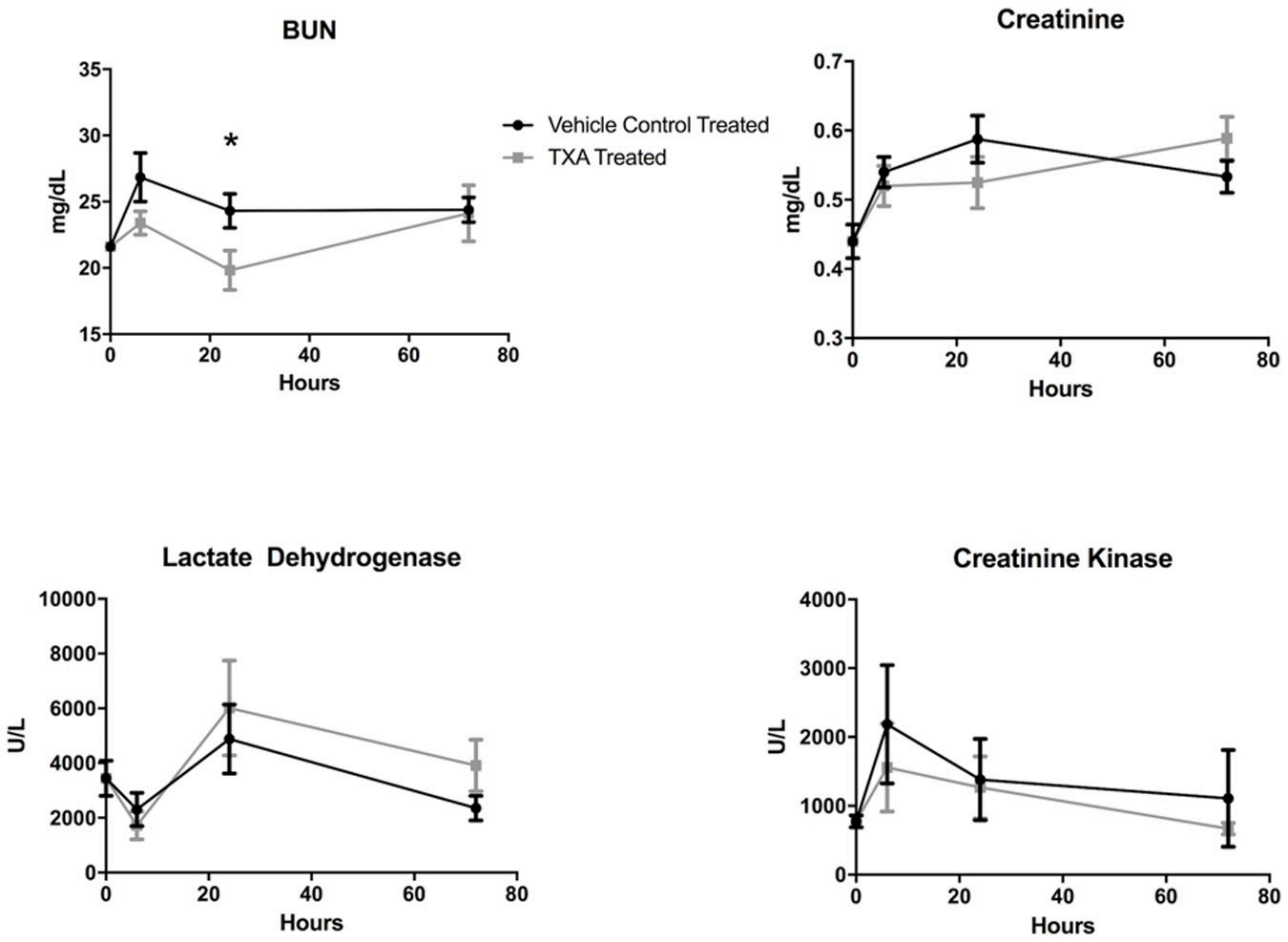
Serum markers of end-organ damage in rats subjected to controlled hemorrhagic shock and resuscitation, vehicle control (normal saline) treated vs. tranexamic acid (TXA) treated, 300mg/kg (*p<0.05, error bars = SEM).

Overall, in both the TXA and vehicle control treatment groups, IL-1β levels were increased at all time points compared to baseline (Fig 3). In the vehicle control treatment group, serum IL-1β peaked at 24 hours after the start of HS. In the TXA treatment arm, serum IL-1β plateaued at six hours and was significantly decreased at 24 hours compared to rats in the vehicle control treatment group (30.2 ± 8.3 vs. 52.5 ± 12.6 pg/mL, p<0.01). Serum IL-10 peaked at 6 hours in the vehicle control treatment group as opposed to at 24 hours in the TXA treatment group. Rats treated with TXA had significantly reduced serum IL-10 levels compared to vehicle control-treated rats at both six (93.8 ± 34.6 vs. 209.7 ± 64.8 pg/mL, p = 0.0002) and 24 hours (109.2 ± 14.7 vs. 166.9 ± 13.9 pg/mL, p = 0.009). HS induced an increase in serum MCP-1 in both treatment arms, with peak production measured at 72 hours post injury. No statistically significant difference in production of MCP-1 was seen between treatment groups. Rats in both treatment arms displayed peak MIP-1α levels at 72 hours post-injury. However, at 24 hours post-injury, MIP-1α expression was significantly reduced in the TXA treatment group 14.5 ± 5.1 pg/mL relative to vehicle control-treated rats (22.7 ± 6.7 pg/mL, p = 0.003).

**Fig 4:**
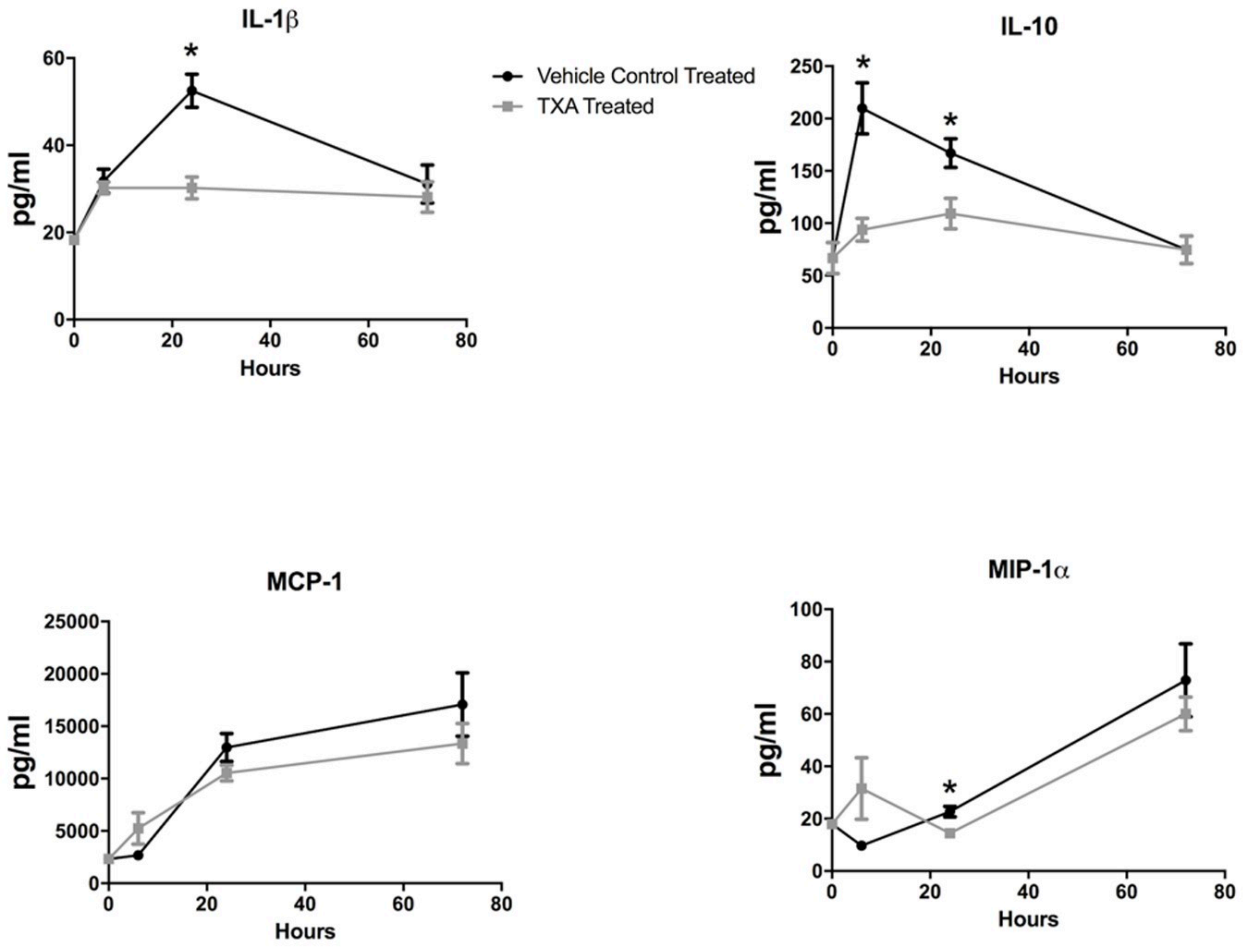
Expression of serum inflammatory cytokines in rats subjected to controlled hemorrhagic shock and resuscitation, vehicle control (normal saline) treated vs. tranexamic acid (TXA) treated, 300mg/kg (*p<0.05, error bars = SEM).

**Fig 5:**
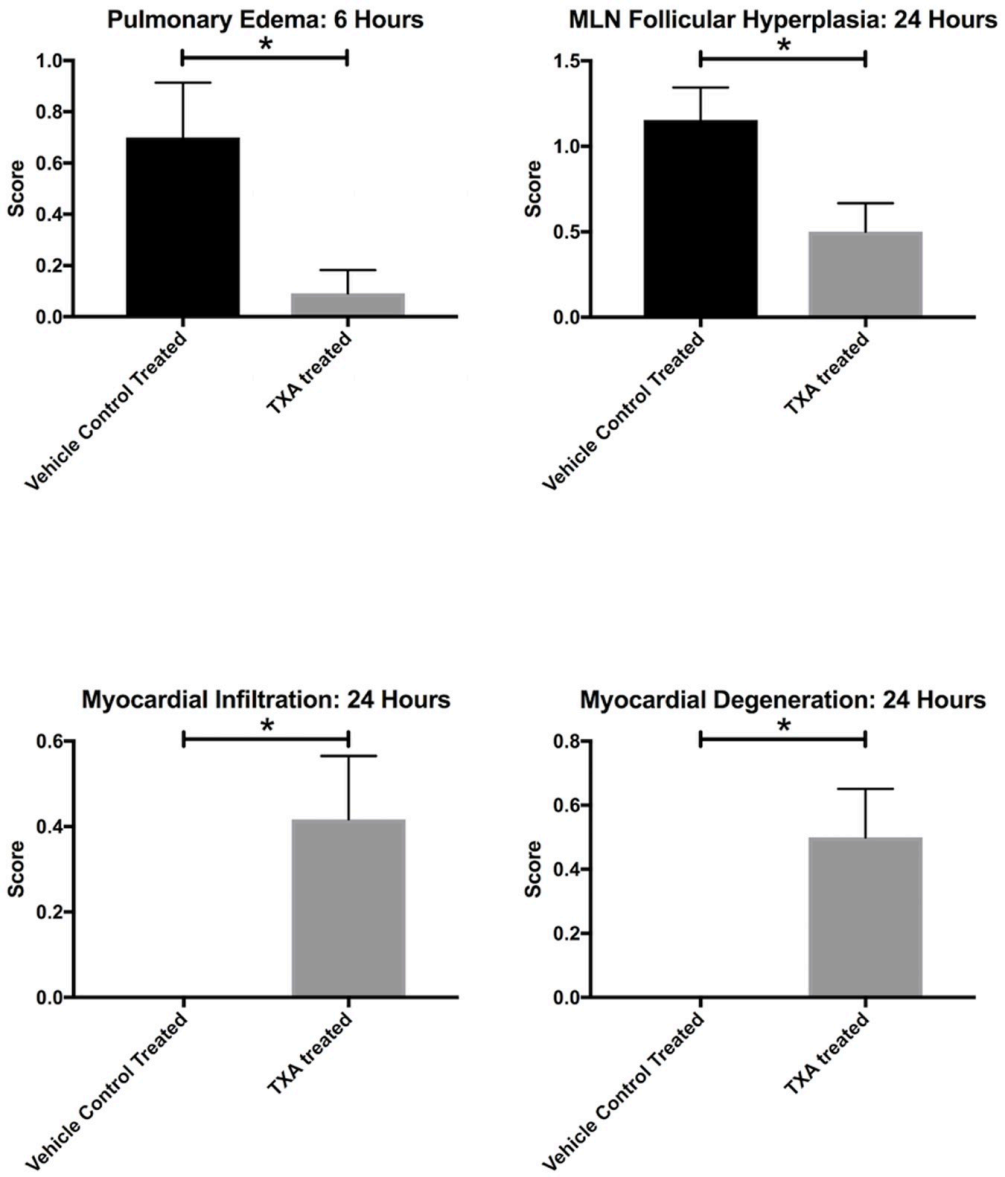
Mean histopathologic scoring of lung, mesenteric lymph node (MLN), and myocardial tissue in rats subjected to controlled hemorrhagic shock and resuscitation, vehicle control (normal saline) treated vs. tranexamic acid (TXA) treated, 300mg/kg (*p<0.05, error bars = SEM).

**Fig 6:**
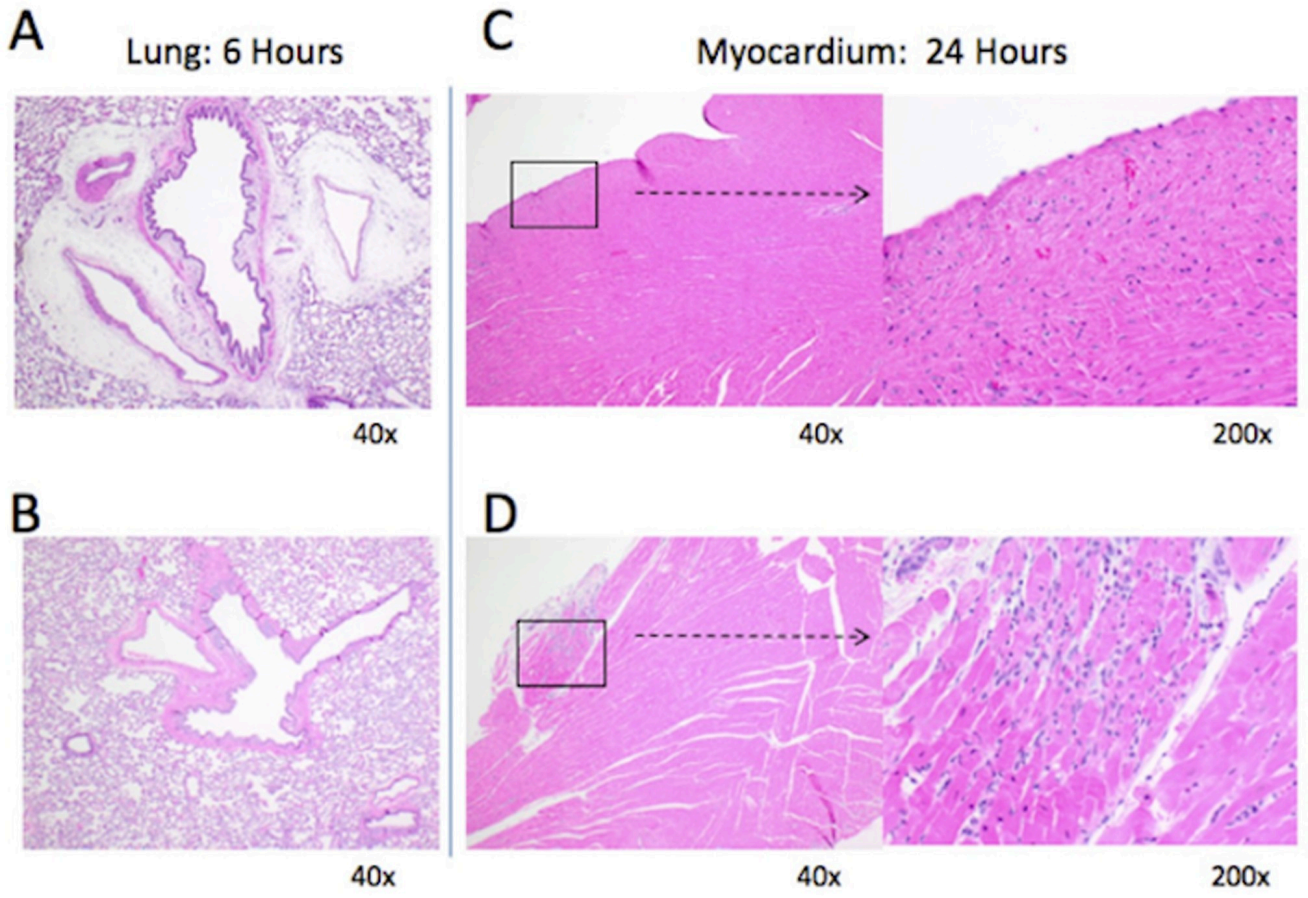
Photomicrographs of lung and myocardial tissue after necropsy in rats subjected to controlled hemorrhagic shock and resuscitation. (**A**) Lung tissue at 6 hours post-injury in vehicle control (normal saline) treated rat with pulmonary perivascular edema present. (**B**) Lung tissue in rat treated with tranexamic acid (TXA) without pulmonary perivascular edema. (**C**) Myocardial tissue at 24 hours post-injury in vehicle control treated rat without myocardial infiltration or degeneration. (**D**) Myocardial tissue in rat treated with TXA with myocardial infiltration and degeneration present.

Histologic analysis showed pulmonary perivascular edema to be significantly reduced in the TXA treatment group at 6 hours post-injury compared to the vehicle control treatment group (mean of 0.1 ± 0.3 vs. 0.7 ± 0.7 on ordinal scale, p = 0.03) (Fig 4). TXA treatment also was associated with decreased follicular hyperplasia in mesenteric lymph nodes at 24 hours post-injury (0.5 ± 0.5 vs. 1.2 ± 0.7, p = 0.04) as shown in Fig 5. However, the TXA treatment group demonstrated significantly increased scores for myocardial lymphocytic infiltration (0.4 ± 0.5 vs. 0 ± 0, p = 0.02) and degeneration (0.5 ± 0.5 vs. 0 ± 0, p = 0.02) at 24 hours compared to controls. Photomicrographs in Fig 6 demonstrate the difference in control (A) and TXA-treated (B) lung tissue and control (C) and TXA-treated (D) myocardium after necropsy. No histologic differences were seen in the liver, kidney, spleen, bowel, muscle, or skin tissues between the TXA and vehicle control treatment groups.

**Fig 7:**
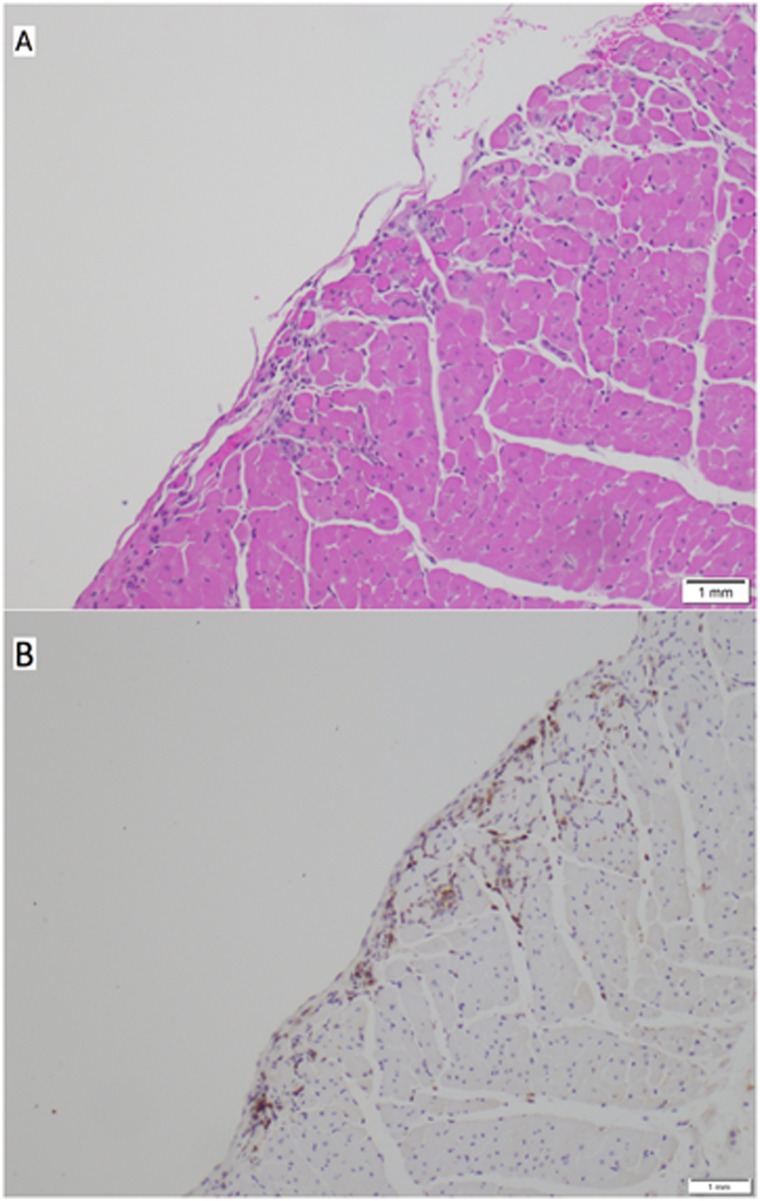
Photomicrographs of myocardial tissue on hematoxylin and eosin staining (A) and on myeloperoxidase (MPO) staining (B) demonstrating neutrophil infiltration.

MPO staining was also performed to evaluate for neutrophil aggregation in pulmonary and myocardial tissue. At six hours post injury, a significant decrease in neutrophil aggregation was observed in pulmonary tissue in animals that had been treated with TXA as compared to the control animals (4230.4 ± 822.6 vs. 6062.8 ± 935.7 neutrophils, p = 0.01). There was also evidence of neutrophil aggregation in myocardial tissue on MPO staining at 24 hours post-injury, as shown in Fig 7. From a quantitative standpoint, however, the MPO staining was inconclusive in the myocardial tissue due to effacement of lesions on the tissue block that had previously been seen on H&E staining.

## Discussion

TXA has been shown to improve mortality in trauma patients. This mortality benefit has been suggested to be related in part to TXA’s anti-inflammatory properties in addition to its anti-fibrinolytic effects, as TXA has not been shown to decrease the need for blood transfusions in this patient population [6,7,19–21]. This study demonstrated a suppressive effect of TXA on IL-1β, IL-10, and MIP-1α expression following HS. TXA was also associated with a decrease in splenic CD8+ T cell counts as well as splenic and peripheral CD4+ T cells. However, any associated end-organ effects between TXA and inflammation were mixed in this study. Pulmonary perivascular edema was decreased with TXA in addition to decreased neutrophil infiltration, suggesting a potential therapeutic benefit. In contrast, there was also evidence of increased myocardial degeneration and necrosis in the treatment arm, possibly attributed to the anti-fibrinolytic properties of TXA.

There are multiple potential mechanisms by which TXA has anti-inflammatory effects. IL-1β was decreased in the TXA group at 24 hours post-injury. IL-1β is associated with a pro-inflammatory cell death process known as pyroptosis and is a vital cytokine in the inflammasome response to injury [22]. There is evidence that TXA decreases nuclear factor κB (NF-κB) activity [23]. As NF-κB is the transcription factor that promotes the expression of pro IL-1β, this may be the mechanism by which TXA decreases IL-1β levels. Additionally, plasmin has been shown to be associated with increased capillary permeability in hyperfibrinolytic states [24]. By inhibiting the cleavage of plasminogen to plasmin, TXA may decrease the amount of capillary leak present. Lastly, TXA in this study appears to reduce the levels of T cells that may play a role in early innate immune responses and not just later cell-mediated responses [25].

A reduction in circulating IL-10 levels was found in the TXA treatment arm of this study. Although IL-10 is a regulatory cytokine in the systemic inflammatory response [26], its systemic production and secretion has been shown to be increased in more severely injured trauma patients with worse outcomes [27]. This suggests that the increased synthesis of IL-10 may be associated with a dysregulated immune system that is contributing to multiorgan dysfunction syndrome (MODS) and could account or increased overall inflammation in the control arm.

This study is consistent with other HS and polytrauma animal models that have shown or suggested a suppressive effect of TXA on inflammation. Wu et al. demonstrated in a polytrauma + HS rodent model that TXA treatment decreased pulmonary edema along with other markers of pulmonary inflammation to include leukocyte and platelet infiltration. In addition, they demonstrated a decrease in lung tissue MCP-1 and migrating monocytes with TXA administration [28]. The authors, however, did not measure serum cytokines in their model, and they euthanized all rats one hour after resuscitation. Thus, there was no short-term period to evaluate the inflammatory response as was done in this study, wherein several key systemic inflammatory mediators were assessed up to 72 hours post-injury. Peng et al. showed that administration of oral TXA to rats following HS resulted in decreased gut and lung inflammation and injury by inhibiting the effects of ADAM-17 and TNF-α on intestinal syndecan-1, which weakens underlying gut mucosa in a typical pro-inflammatory state [29]. No notable changes in gut histology were seen in this current study, however TXA was administered intravenously as opposed to orally. Although the benefit of TXA is associated in large part with its antifibrinolytic properties, Roy and colleagues demonstrated a survival benefit associated with TXA in rats in HS despite the absence of fibrinolysis measured on rotational thromboelastometry [30]. Their findings support the hypothesis of an alternative mechanism, other than through anti-fibrinolysis, by which TXA provides a survival benefit.

Other studies have shown little to no effects of TXA on serum inflammatory markers. Boudreau et al demonstrated a marked increase in serum levels of IL-6, MCP-1, MIP-1α, and RANTES when HS to a systolic blood pressure of 25mmHg for one hour was added to a murine closed traumatic brain injury (TBI) model [31]. However, there was no difference in the circulating level of these inflammatory markers with TXA administration. Of note, the authors administered an intraperitoneal injection of TXA as opposed to the standard intravenous or oral versions of TXA. Additionally, they gave a TXA dose of 10 mg/kg, which is less than the 300 mg/kg dose used in this study.

With regard to end-organ injury, the decreased pulmonary edema and neutrophil infiltration observed in this study with TXA treatment is consistent with previous findings which report that TXA mitigates acute lung injury, including lung edema [28]. Although post-HS mesenteric lymph has been shown to play a role in acute lung injury and acute kidney injury in these patients [32,33], it is unclear if the decreased mesenteric lymph node follicular hyperplasia observed in the current TXA study has a role in decreased systemic inflammation given that these lesions can often take more than 72 hours to appear. The increase in the peripheral neutrophil count in the TXA group at 6 hours post-injury was unexpected. This perhaps represents a quicker demargination in the TXA group as the neutrophil counts were essentially the same in the two groups at 24 and 72 hours post-injury. Outside of only a transient decrease in BUN at 24 hours in the TXA treatment group, there were not other laboratory or histologic changes suggesting renal changes associated with TXA treatment.

In this study, TXA administration was associated with increased myocardial necrosis and degeneration as well as inflammatory cell infiltration. Myocardial infarction is an infrequent but noted potential complication of TXA [34]. It is therefore possible that our results are associated with its anti-fibrinolytic properties. With regard to the increased myocardial infiltrate seen with TXA administration, an association between lymphocytic myocardial infiltration and myocardial infarction has been found in a study of human hearts at autopsy [35]. Despite these histologic findings, however, a difference in CK levels between the two treatment groups was not demonstrated. Thus, while histologically evident, a physiologically relevant degree of myocardial degeneration and necrosis is not supported by the laboratory data from this rat model. Troponin testing may have been a more sensitive clinic marker for myocardial ischemia compared to CK for this study.

Interestingly, in the CRASH-2 trial, patients that were administered TXA had a lower incidence of post-traumatic myocardial infarction compared to those that did not receive the drug [36]. Likewise, in the MATTERs study, TXA was not found to be an independent predictor for thromboembolic complications [7]. However, high dose TXA has been shown to be associated with an increased risk of seizures in cardiac surgery patients [5,37]. Given these disparate findings, more information is needed regarding potential adverse effects associated with widespread administration of TXA in trauma patients.

There are several limitations present in this study. Pre-designated euthanasia endpoints were established. It was therefore not possible to determine if TXA was associated with a mortality difference. Coagulation parameters were not evaluated, although this was not the focus of this study. Thus, it is certainly possible that the potential benefits of TXA may be directly related to its anti-fibrinolytic effects. Compared to other rodent studies, a larger dose of TXA was used in this study—the maximum allowable dose without known adverse effects. Future studies evaluating TXA on inflammation in larger animal models as well as if delayed administration influences the inflammatory profile will further develop our understanding on the immunomodulatory effects of TXA in trauma.

In conclusion, this study demonstrated that TXA is associated with an overall suppressive effect on a few key inflammatory mediators in a rodent model of controlled HS. In addition, while TXA treatment was associated with reduced pulmonary edema and mesenteric lymph node follicular hyperplasia, it was also associated with increased myocardial degeneration, which is potentially related to the pro-thrombotic effect of TXA. Further studies are needed to better understand which patients may be at most risk for adverse effects of TXA and which patients may benefit the most from the potential immunomodulatory as well as the anti-fibrinolytic effects of TXA in trauma.
